# Field survey of Cassidinae beetles (Coleoptera, Chrysomelidae) and their host plants in southern Guangxi, China

**DOI:** 10.3897/BDJ.11.e107523

**Published:** 2023-07-31

**Authors:** Chaokun Yang, Chengqing Liao, Jiasheng Xu, Peng Liu, Charles L. Staines, Xiaohua Dai

**Affiliations:** 1 Leafminer Group, School of Life Sciences, Gannan Normal University, Ganzhou, China Leafminer Group, School of Life Sciences, Gannan Normal University Ganzhou China; 2 Smithsonian Environmental Research Center, Edgewater, United States of America Smithsonian Environmental Research Center Edgewater United States of America; 3 National Navel-Orange Engineering Research Center, Ganzhou, China National Navel-Orange Engineering Research Center Ganzhou China; 4 Ganzhou Key Laboratory of Nanling Insect Biology, Ganzhou, China Ganzhou Key Laboratory of Nanling Insect Biology Ganzhou China

**Keywords:** Cassidinae, host plant, faunal composition, insect-plant network, food web, bipartite network

## Abstract

Few systematic studies have been conducted on the faunal composition and food web structure of Cassidinae of China. During 2013-2019, we systematically investigated Cassidinae beetles and their host plants in the southern Guangxi. A total of 2,255 Cassidinae individuals from 66 species, 23 genera and ten tribes were collected in southern Guangxi. Most species belonged to the tribe Hispini (23 species, 34.8%), followed by the tribe Gonophorini (13 species, 19.7%), Cassidini (eight species, 12.1%) and Aspidimorphini (six species, 9.1%). The others (16 species) belonged to the tribes Anisoderini, Botryonopini, Callispini, Oncocephalini, Notosacanthini and Leptispini. The tribe Notosacanthini was recorded from Guangxi for the first time. The genera *Neownesia* (Botryonopini), *Gonophora* (Gonophorini), *Micrispa* (Gonophorini), *Notosacantha* (Notosacanthini) and *Prionispa* (Oncocephalini) were firstly recorded in Guangxi. In total, we obtained 47 newly-recorded species in southern Guangxi and 33 newly-recorded species in the whole Guangxi, of which, *Callispafrontalis* Medvedev, 1992 was newly recorded in China. *Dactylispafeae* Gestro (625 individuals) and *D.chinensis* Weise (565 individuals) were the most common species. A total of 69 species, 53 genera and 19 families of host plants were identified for Cassidinae in southern Guangxi. Many host plant associations are new records for Cassidinae. Quantitative food web analysis indicated that Cassidinae species in southern Guangxi primarily fed on Poaceae, Convolvulaceae, Cyperaceae and Rosaceae. Generally, the plant-Cassidinae food webs were moderately complex and stable in southern Guangxi. This is the first large contribution to the knowledge of the species composition and host plant diversity of Cassidinae in southern Guangxi.

## Introduction

Cassidinae *s.l.* is the second largest subfamily in Chrysomelidae (leaf beetles), with 6,273 species, 339 genera and 35 tribes (Chen et al. 1986, Staines 2015, Hobern et al. 2021, Borowiec and Świętojańska 2022). The subfamily consists of hispine beetles (formerly ‘Hispinae’) and tortoise beetles (formerly Cassidinae *s.s.*) (Staines 2002, Chaboo 2007).

Few systematic studies have been conducted on Cassidinae community composition and host diversity in China (Liu et al. 2019). Currently, 478 species have been recorded in China (Chen et al. 1986, Hua 2002, Qi et al. 2008, Staines 2015, Liao et al. 2018, Borowski 2020, Borowiec and Świętojańska 2022), accounting for 7.6% of the world’s Cassidinae richness. However, through our fieldwork in recent years, we predict a much higher Cassidinae richness yet to be discovered in China because Cassidinae is poorly studied in some Chinese biomes and regions.

Southern Guangxi (abbreviation for the southern region of Guangxi Zhuang Autonomous Region, China) belongs to the south-subtropical monsoon climate zone, with abundant rainfall and heat (Qin et al. 2011, Tan et al. 2014). Southern Guangxi has typical tropical forests. It hosts rich plant and animal species, with some national and autonomous region-protected ones (*Camelliapetelotii* (Merrill) Sealy, *Parashoreachinensis* Wang Hsie, *Alsophilaspinulosa* (Wall. ex Hook.) R. M. Tryon, *Aquilaheliacal* Savigny and *Trachypithecusleucocephalus* Tan) (Department of Forestry of Guangxi Zhuang Autonomous Region 1993, Qin et al. 2011). It also has 23 nature reserves (nine national, twelve autonomous regions, one city and one county) and four national forest parks (Ministry of Ecology and Environment of the People's Republic of China 2019, Department of Forestry of Guangxi Zhuang Autonomous Region 2022). Therefore, it is considered one of the biodiversity hotspots in China.

In southern Guangxi, some comprehensive investigations were conducted on the biodiversity of plants and higher animals (Department of Forestry of Guangxi Zhuang Autonomous Region 1993, Huang and Nong 2002, Guangxi Forest Inventory & Planning Institute and Guangxi Shiwandashan Nature Reserve Administration 2002). Preliminary lists of common insects have also been conducted in some regions (Lu 2013, Wei et al. 2014, Zhang et al. 2014, Yang et al. 2015, Zhao and Liu 2015, Yang et al. 2016, Wan et al. 2020, Hu et al. 2021). However, few systematic studies of the insect fauna and community composition have been reported (Mo et al. 2014, Zhang et al. 2014, Gu et al. 2019, Hu et al. 2021). There are some reports on Cassidinae in southern Guangxi (Chen et al. 1986, Ye 2005, Pang et al. 2012, Mo et al. 2014). However, most of these Cassidinae checklists are far below expectation, without deep analysis of their faunal composition and food web structure. Since 2013, we have carried out several investigations in southern Guangxi to obtain first-hand information on Cassidinae and their host plants. The results can provide a theoretical basis for further research on insect diversity in Guangxi and its forest protection and pest control.

## Material and methods

### Study area

Southern Guangxi, including the cities of Nanning, Chongzuo, Qinzhou, Fangchenggang and Beihai, are located in south China (Fig. 1). During 2013-2019, Cassidinae and their host plants were surveyed and collected in six areas of southern Guangxi: Damingshan National Nature Reserve together with Longshan Autonomous Region Nature Reserve (Damingshan) (Fig. 2b, c) (Zhou et al. 2009, Zhou and Zhou 2013, Tan et al. 2014, Yang et al. 2016), Fangcheng Golden Camellia National Nature Reserve (Fangcheng) (Tan et al. 2014, Pan et al. 2016, Wu et al. 2022), Nonggang National Nature Reserve (Nonggang) (Fig. 2a) (You and Li 1988, Huang et al. 2013, Wang et al. 2016), Shiwandashan National Nature Reserve (Shiwandashan) (Fig. 2d) (Lu et al. 1995, Tan et al. 2014, Hu et al. 2021), Wuhuangshan National Geopark (Wuhuangshan) (Wuhuang Mountain National Geopark 2017) and Xidamingshan Autonomous Region Nature Reserve together with Longhushan Autonomous Region Nature Reserve (Xidamingshan) (Yang 2013, Tan et al. 2014) (Suppl. material 1). No Cassidinae beetles were found in Nongla Autonomous Region Nature Reserve and the urban parks in Nanling City (Fig. 1b).

### Specimen collection and identification

Based on digital maps, our previous experience and the knowledge of local guides, we chose survey paths through suitable habitats. Survey hours were usually between 8:30 am and 4:00 pm. Potential host plants were carefully investigated for Cassidinae adults or larvae. Both plants and beetles were placed in plastic zip-lock bags. Collection information including date, location and altitude were recorded. In the laboratory, plants and Cassidinae-fed leaves were individually scanned into images using an Epson 10000XL scanner (Dai et al. 2018) for further confirmation of Cassidinae-plant associations. Cassidinae larvae or pupae were raised to adults in the laboratory. Some adults (799 individuals) were treated with 99.5% ethyl acetate (AR) and pinned with minuten pins. The others (1,456 individuals) were preserved in 99.8% ethanol (GR) at -80°C for future DNA sequencing. Photos of Cassidinae individuals were taken with a SONY A7RIV+LAOWA 25 mm. Cassidinae species were preliminarily identified under an Olympus stereomicroscope SZX16, according to two keys (Chen et al. 1986, Staines 2012) and an interactive manual for world tortoise beetles (Borowiec and Świętojańska 2022). All Cassidinae species were finally confirmed by Dr. Lukáš Sekerka (the Department of Entomology, National Museum, Natural History Museum, 1740 Cirkusová, Czech Republic). Host identities of collected plants were confirmed by their larval or adult feeding damage (Liu et al. 2019). Plant species were identified by Dr. Xiaoya Yu (Qiannan Normal University for Nationalities) and Dr. Yinghua Luo (Guangxi University). All specimens of Cassidinae beetles were stored in the Nanling Herbarium, Gannan Normal University (GNNU).

### Data analysis

Scientific names of all species (including beetles and hosts) are checked on the Catalogue of Life website (https://www.catalogueoflife.org) (Hobern et al. 2021) and Global Biodiversity Information Facility website (GBIF) (https://www.gbif.org/) (GBIF Secretariat 2022). A checklist of Cassidinae and their host plants in southern Guangxi was obtained. Faunal composition analyses were carried out in Microsoft Excel 2016.

In order to better present the association between Cassidinae beetles and their host plants, the food webs of plant family-Cassidinae tribe and also that of plant family-Cassidinae genus were constructed using the "bipartite" Package (Dormann et al. 2008, Dormann et al. 2009, Dormann 2011) with R 4.3.0 (R Core Team 2023) in the graphic user interface of RStudio (RStudio Team 2023) . Quantitative food web metrics in this study were listed in Table 1. The values of plant-Cassidinae matrices used in the above analyses were the number of Cassidinae species feeding on one plant family.

The map was constructed using QGIS 3.26.3 (QGIS Development Team 2022) . QGIS MapTiler Plugin can obtain OpenStreetMap data from the OpenMapTiles project (openstreetmap.org) through the MapTiler Cloud under the Open Database License.

## Results

### Faunal composition in southern Guangxi

All individuals were identified to the species level, except for 123 individuals, which were identified to the genus level (Table 2, Suppl. material 2). A total of ten tribes (Figs 3, 4), 23 genera, 66 species and 2,255 individuals of Cassidinae were collected. Notosacanthini was recorded in Guangxi for the first time. *Callispafrontalis* Medvedev, 1992 was newly recorded in China.

The tribes with the highest number of genera were Gonophorini (six genera) and Hispini (four genera), together accounting for 43.5% of the total, followed by Cassidini (three genera), Aspidimorphini (two genera), Oncocephalini (two genera) and the tribes Anisoderini, Botryonopini, Callispini, Notosacanthini and Leptispini (one genus each).

The most species-rich tribe was Hispini (23 species), followed by Gonophorini (13 species), together accounting for 54.5% of the total. The tribes Cassidini (eight species) and Aspidimorphini (six species). The remaining 16 species belonged to six tribes (Callispini [5], Oncocephalini [3], Anisoderini [3], Notosacanthini [2], Leptispini [2] and Botryonopini [1]).

**Figure 5.**
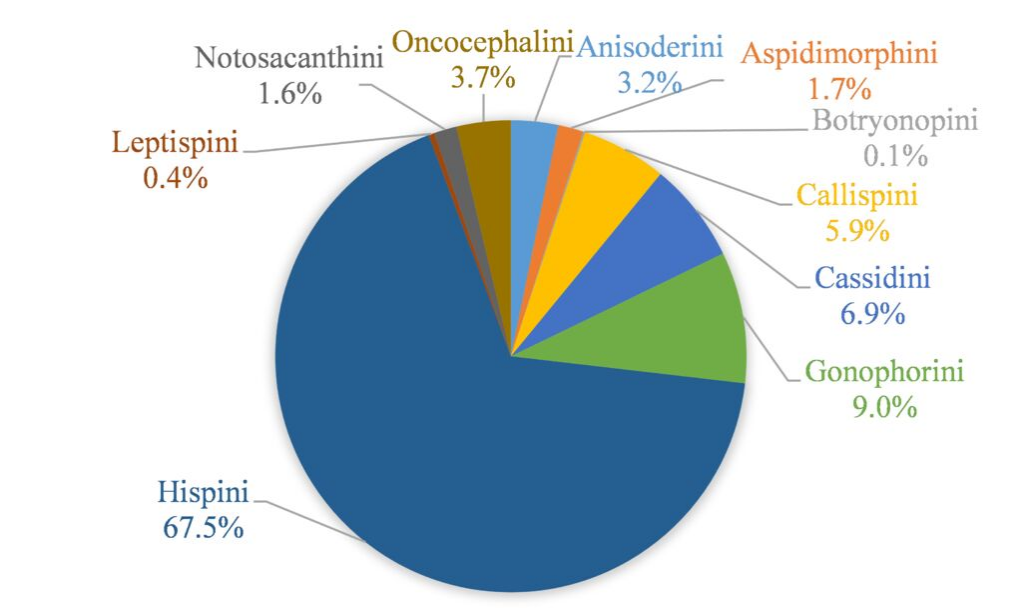
Percentage distribution of individuals numbers in the ten Cassidinae tribes.

The tribe Hispini (1,522 individuals) had the highest number of individuals, with 67.5% of the total, followed by Gonophorini (9%, 203 individuals), Cassidini (6.9%, 155 individuals) and Callispini (5.9%, 132 individuals). The remaining 242 individuals belonged to six tribes (Anisoderini, Aspidimorphini, Botryonopini, Oncocephalini, Notosacanthini and Leptispini) (Fig. 5). At the species level, *Dactylispafeae* Gestro (625 individuals) and *D.xanthopus* Gestro (565 individuals) were the most common species.

In Hispini, *Dactylispa* Weise was the dominant genus in both species and individuals, with 20 species (87% of total) and 1,477 individuals (97% of total), while *Asamangulia* Maulik (18 individuals), *Hispa* Linnaeus (25 individuals) and *Rhadinosa* Weise (two individuals) had one species each.

In Gonophorini, there were 203 individuals from 13 species and six genera. The most abundant genus was *Agonita* Strand, with four species (30.8% of the total) and 79 individuals (38.9% of the total) and then *Downesia* Baly, with four species (30.8%) and 69 individuals (34%). Other genera were *Klitispa* Uhmann (39 individuals) with two species, *Sinagonia* Chen et T'an (ten individuals), *Gonophora* Chevrolat (four individuals) and *Micrispa* Gestro (two individuals) with one species each.

In Cassidini, the specimens (155 individuals) belonged to three genera (*Cassida* Linnaeus, *Chiridopsis* Spaeth and *Thlaspida* Weise). *Cassida* Linnaeus had six species (75%) and 66 individuals (42.3%), *Chiridopsis* Spaeth (13 individuals) and *Thlaspida* Weise (76 individuals) had one species each.

### Newly-recorded Cassidinae beetle species

**Figure 6.**
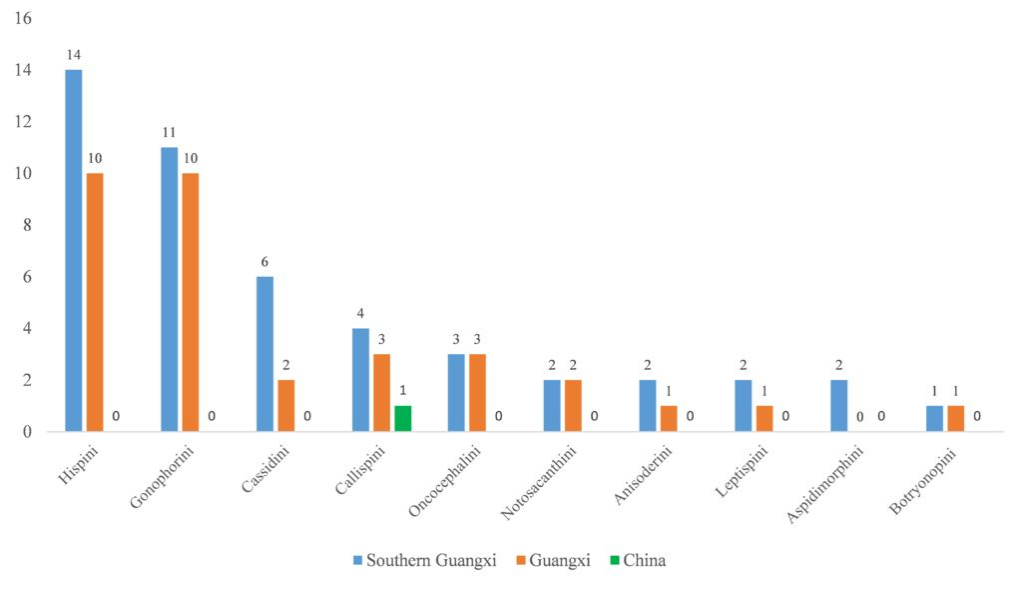
Frequency distribution of newly-recorded species amongst different Cassidinae tribes in southern Guangxi, Guangxi or China.

Sixty-six species of Cassidinae were collected, with 47 newly-recorded species in southern Guangxi, 33 newly-recorded species in the whole Guangxi and one newly-recorded species in China (Fig. 6).

Except for the tribe Aspidimorphini, the remaining nine tribes had newly-recorded species in Guangxi. The tribe with the greatest number of newly-recorded species was Hispini, with 14 new records in southern Guangxi and ten in Guangxi. The tribe Gonophorini had eleven newly-recorded species in southern Guangxi and ten in Guangxi. The tribe Cassidini had six newly-recorded species in southern Guangxi and two in Guangxi. The tribe Callispini had four newly-recorded species in southern Guangxi and three in Guangxi, amongst which, *Callispafrontalis* Medvedev, 1992 was newly recorded in China. The tribe Oncocephalini had three newly-recorded species in southern Guangxi as well as in Guangxi. The tribe Notosacanthini was recorded in Guangxi for the first time and it had two newly-recorded species in southern Guangxi as well as in Guangxi. The tribes Anisoderini and Leptispini had two newly-recorded species in southern Guangxi and one in Guangxi, respectively. The tribe Botryonopini had one newly-recorded species in southern Guangxi as well as in Guangxi.

### The host plant-Cassidiane food web in southern Guangxi

A total of 19 families, 53 genera and 69 species of host plants of Cassidinae were collected in southern Guangxi. The family Poaceae had the richest hosts (29 species), followed by Rubiaceae (seven host species), Rosaceae (four host species) and then Cyperaceae, Convolvulaceae, Lamiaceae, Phyllanthaceae and Zingiberaceae (each with three host species). Many host plant associations were new records for Cassidinae (Suppl. material 3).

In the two quantitative food webs between host plant families and Cassidinae groups in southern Guangxi (Table 3), both links per taxa and connectance were low, indicating that there were many missing links between plant families and the Cassidinae group. The generality values showed that each Cassidinae tribe averaged about 4.0 host plant families, while each Cassidinae genus had 3.6. The vulnerability values showed that each plant family might, on average, feed 2.7 Cassidinae tribes and 4.8 Cassidinae genera, respectively. In both food webs, the values of specialisation degree, weighted nestedness and robustness were moderate (Table 3).

**Figure 7.**
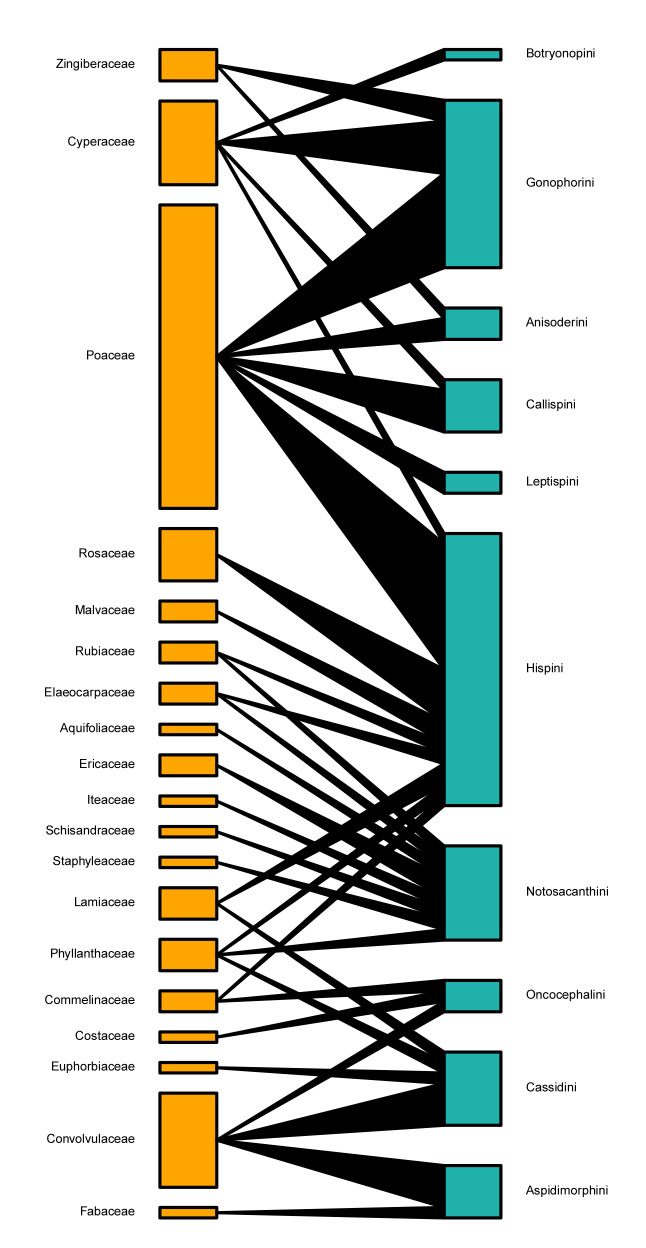
Quantitative food web between 19 host plant families (in yellow) and ten Cassidinae tribes (in blue).

**Figure 8.**
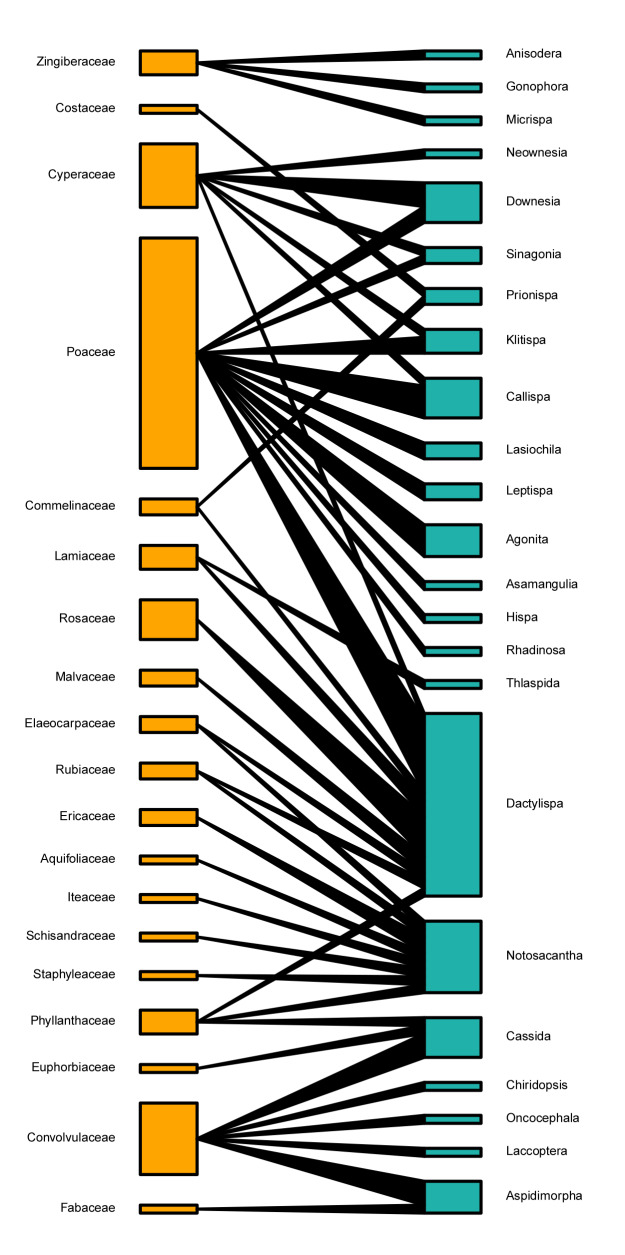
Quantitative food web between 19 host plant families (in yellow) and 23 Cassidinae genera (in blue).

According to the food web plots between plant families and Cassidinae groups (Figs 7, 8), Poaceae, Convolvulaceae, Cyperaceae and Rosaceae were fed on by the highest number of Cassidinae species, which consisted of five tribes and twelve genera, four tribes and six genera, three tribes and five genera, one tribe and one genera, repectively. In the food web at Cassidinae tribal level, Hispini fed on the largest number of host plant families at nine. Notosacanthini had eight host plant families, of which Aquifoliaceae, Elaeocarpaceae, Iteaceae, Schisandraceae and Staphyleaceae are new host records. Both Botryonopini and Leptispini each fed on only a single host plant family (Fig. 7). In the food web at Cassidinae generic level, *Dactylispa* Weise had the largest number of host plant families at nine, followed by *Notosacantha* Chevrolat, with eight host plant families. (Fig. 8)

## Discussion

This study is the first systematic investigation of the community composition and species abundance of Cassidinae in southern Guangxi. Sixty-six Cassidinae species were collected, which was 13.8% of the total number of Cassidinae species known from China. At the tribal level, Hispini had the highest number of species in southern Guangxi (23 species, 16.9% of China fauna). At the generic level, *Dactylispa* Weise had the highest number of species (20 species, 14.7% of China), In our previous report in Longnan County, Hispini is also the most dominant tribe, while *Dactylispa* Weise is also the most dominant genus (Liu et al. 2019).

Amongst the six sites, ten tribes, 20 genera, 55 species and 2053 individuals were collected at Damingshan; six tribes, eight genera, twelve species and 42 individuals were collected at Nonggang; five tribes, eight genera, twelve species and 110 individuals were collected at Xidamingshan; three tribes, four genera, seven species and 41 individuals were collected at Shiwandashan; one tribe, one genus, three species and eight individuals were collected at Wuhuangshan; and only one species and one individual were collected at Fangcheng. Damingshan had the highest number of Cassidinae species and individuals, followed by Nonggang, Shiwandashan and Xidamingshan, while the lowest number of species and individuals were found in Wuhuangshan and Fangcheng. Such variation of cassidinae composition in different places, might be related to several possible reasons such as different climatic conditions, different plant richness, different human disturbance levels and different sampling efforts.

Forty-four Cassidinae species have been previously reported in southern Guangxi (with a terrestrial area of 43,984,900 hm^2^) (Chen et al. 1986, Lin 1992, Mo et al. 2014). Compared to the pantropical regions, such as Hainan Province (51 species/3,540,000 hm^2^) and Xishuangbannan, Yunnan Province (186 species/1,909,600 hm^2^) (Chen et al. 1986), the Cassidinae richness in southern Guangxi may be underestimated. Our findings have nearly doubled the number of Cassidinae species to 66. Amongst them, 47 species were newly recorded in southern Guangxi, 33 species were newly recorded in Guangxi and one newly-recorded species in China. *Callispafrontalis* Medvedev, 1992 was native to Vihnfou, Vietnam (Staines 2015) and is now discovered in China. During our recent investigations on Cassidinae in Longnan County, we have found 59 species, 16 genera and eight tribes (Liu et al. 2019), with 38 species, twelve genera and seven tribes in Jiulianshan National Nature Reserve. However, only seven species, six genera and five tribes have previously been reported in the same Reserve (Zhang 1987, Liu et al. 2002). Cassidinae fauna are still poorly known in many regions of China.

Generally, the plant-Cassidinae food webs in southern Guangxi were moderately complex and stable. Similar to the previous study on a neotropical host plant-hispine beetle food web (Meskens et al. 2011), connectance values in this study were also smaller than 0.2, indicating small propotions of the total potential number of interactions. The nestedness in both studies was moderate (Meskens et al. 2011). Host plants of Cassidinae are in diverse plant taxonomic groups and life forms, and different Cassidinae groups may prefer different plant groups (Chen et al. 1986, Buzzi 1994, Hawkeswood 2003, Chaboo 2007, Sultan et al. 2008, Liao et al. 2015, Liao 2015, Staines 2015, Maican and Serafim 2017, Reid 2017, Liu 2018, Liu et al. 2019, Morrison et al. 2019, Nishida et al. 2020, Ranade et al. 2021, Schnepp and Riley 2021, Gomes et al. 2021, Özdikmen and Coral Şahin 2021, Santos Cajé et al. 2021, Borowiec and Świętojańska 2022). Considering the leaf-mining hispines around the world, their hosts cover 35 plant orders, 80 plant families and 443 plant genera, most of which belong to the Poaceae (Liao et al. 2015). Poaceae was also the dominant plant family in our study, with 29 host species, accounting for 42% of the total host plants. Our results are consistent with previous reports that Poaceae has the richest hosts for Cassidinae (Chen et al. 1986, Liu et al. 2019). One single Cassidinae species may feed on diverse plants and the host ranges of Cassidinae larvae are generally narrower than those of their adults. For example, *Dicladispaarmigera* Olivier can feed on eight plant families and 42 plant species (Liao et al. 2015, Staines 2015), but its larvae can only complete the life cycle on the genera *Oryza* L. and *Zizania* L. (Poaceae) (Chen et al. 1986). *Dactylispaangulosa* Solsky can feed on seven families and 20 species of plants (Chen et al. 1986, Staines 2015, Borowski 2020). We also found that seven Cassidinae beetles, including *Anisoderafraterna* Baly, *Downesiatarsata* Baly, *Dactylispafeae* Gestro, *D.longispina* Gressitt, *D.sauteri* Uhmann, *Hispaandrewesi* Weise and *Notosacantha* sp., could feed on five or more host species in southern Guangxi. On the other hand, different Cassidinae species can utilise the same host plant. For example, *Ipomoeabatatas* (L.) Lamarck (Convolvulaceae) can be fed on by 73 Cassidinae species, (e.g. *Cassidacircumdata* Herbst, *Charidotellaflaviae* Maia et Buzzi, *Glyphocassistrilineata* Hope, Laccoptera (Laccopteroidea) nepalensis Boheman and *Aspidimorpha* (*s. str.*) *miliaris* Fabricius) (Chen et al. 1986, Buzzi 1994, Sultan et al. 2008, Hugh and Heron 2011, Fernandes and Linzmeier 2012, Reddy 2015, Morrison and Windsor 2017, Morrison et al. 2019, Dube et al. 2020, Huang 2020, Toledo-Perdomo 2020, Berasategui et al. 2022, Borowiec and Świętojańska 2022, Pons et al. 2022). In our investigation, nineteen plant species (e.g. *Carexcruciata* Wahlenb. (Cyperaceae), *Lophatherumgracile* Brongn. (Poaceae), *Rubuscochinchinensis* Tratt. (Rosaceae) and Merremiaumbellatesubsp.orientalis (H.Hallier) van Ooststroom (Convolvulaceae)) were fed on by three or more Cassidinae species. In our analyses of the host family-Cassidinae food web, at the tribal level, five Cassidinae tribes feed on monocotyledons, three tribes feed on dicotyledons, while Hispini and Oncocephalini can feed on both monocotyledons and dicotyledons (Fig. 7). At the generic level, most Cassidinae genera feed on monocotyledonous plants, while only *Dactylispa* Weise can feed on both monocotyledonous and dicotyledonous plants (Fig. 8). In general, the Cassidinae beetles we collected in southern Guangxi mainly feed on monocotyledonous plants, which is consistent with previous reports on the host composition of Cassidinae (Chen et al. 1986, Chaboo 2007, Staines 2015).

The results of this study may reflect only a tiny part of the insect resources in southern Guangxi. Due to the limitations of working time and human resources, we only collected specimens from some representative protected areas. In addition, some specimens might be damaged during collection, transportation, raising and preservation. Therefore, some Cassidinae species still lacked information on their host plants and life histories. In the future, continuous in-depth investigations are still needed to reveal the diversity and ecology of Cassidinae.

## Supplementary Material

217C42B5-2355-53F8-9F1F-373F5B711608 10.3897/BDJ.11.e107523.suppl1 8207563 Supplementary material 1 Background information of our survey sites in southern Guangxi, Guangxi Zhuang Autonomous Region, China Data type Geographic locations and environmental factors Brief description Geographic, climatic and biological information of the six collection sites. File: oo_885309.xlsx https://binary.pensoft.net/file/885309 Chaokun Yang, Chengqing Liao, Jiasheng Xu, Peng Liu, Charles L. Staines, Xiaohua Dai

7DDC032A-1054-5F08-834F-CA8DC6ED5BC5 10.3897/BDJ.11.e107523.suppl2 Supplementary material 2 Cassidinae beetles and their confirmed host plants in southern Guangxi, Guangxi Zhuang Autonomous Region, China Data type Checklist, taxonomic status, insect-plant associations and distribution sites Brief description All identified Cassidinae beetles and their confirmed host plants, including the occurrences of Cassidinae species in six collection sites. File: oo_885311.xlsx https://binary.pensoft.net/file/885311 Chaokun Yang, Chengqing Liao, Jiasheng Xu, Peng Liu, Charles L. Staines, Xiaohua Dai

DC9D0F5D-5918-5DA4-B8EC-52D70821AC12 10.3897/BDJ.11.e107523.suppl3 Supplementary material 3 Host plants and their corresponding Cassidinae beetles in southern Guangxi, Guangxi Zhuang Autonomous Region, China Data type Checklist, taxonomic status, insect-plant associations and distribution sites Brief description Associations between host plants and their corresponding Cassidinae beetles. File: oo_885312.xlsx https://binary.pensoft.net/file/885312 Chaokun Yang, Chengqing Liao, Jiasheng Xu, Peng Liu, Charles L. Staines, Xiaohua Dai

## Figures and Tables

**Figure 1a.**
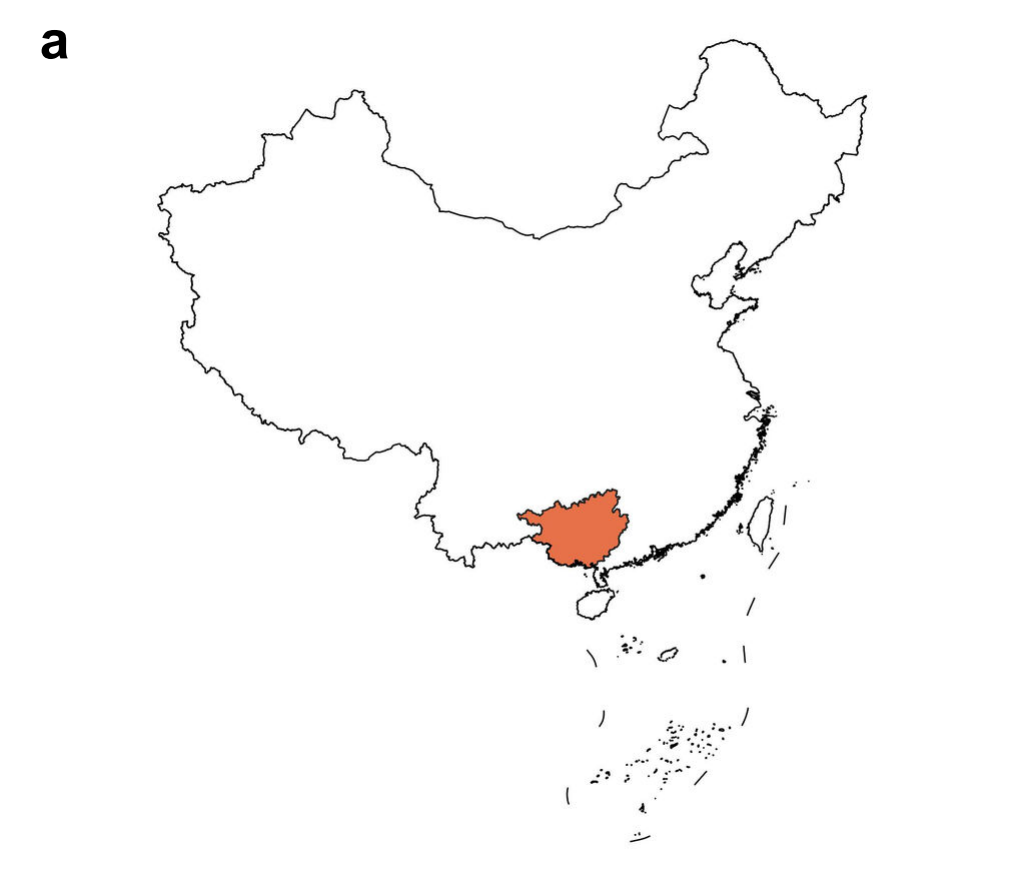
Guangxi;

**Figure 1b.**
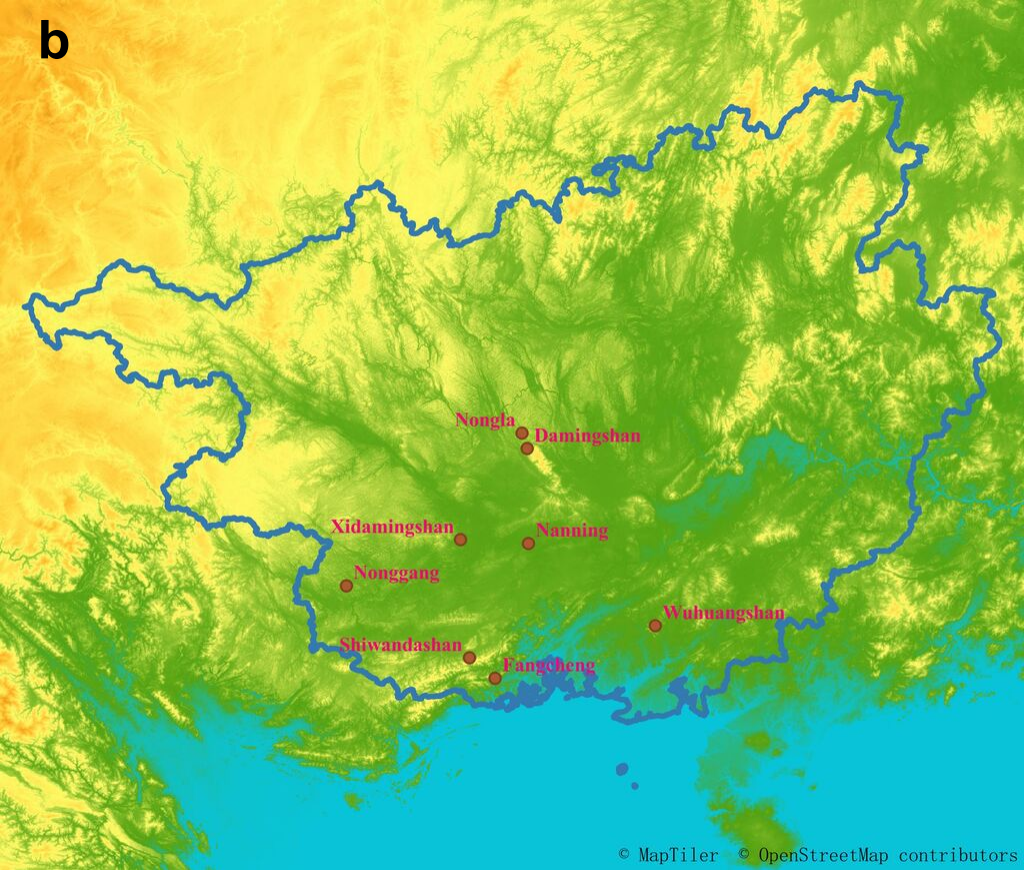
Survey sites.

**Figure 2a.**
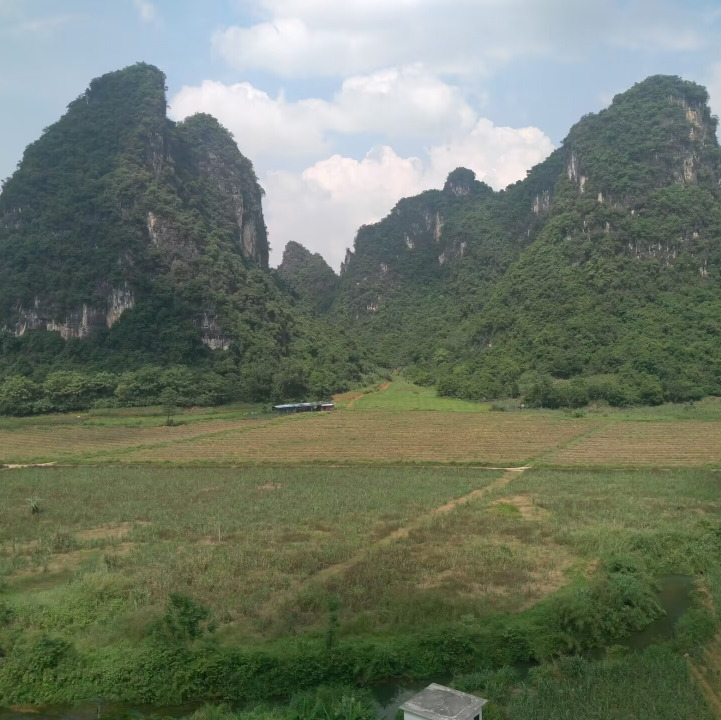
Nonggang (Photo by Lixin Cui);

**Figure 2b.**
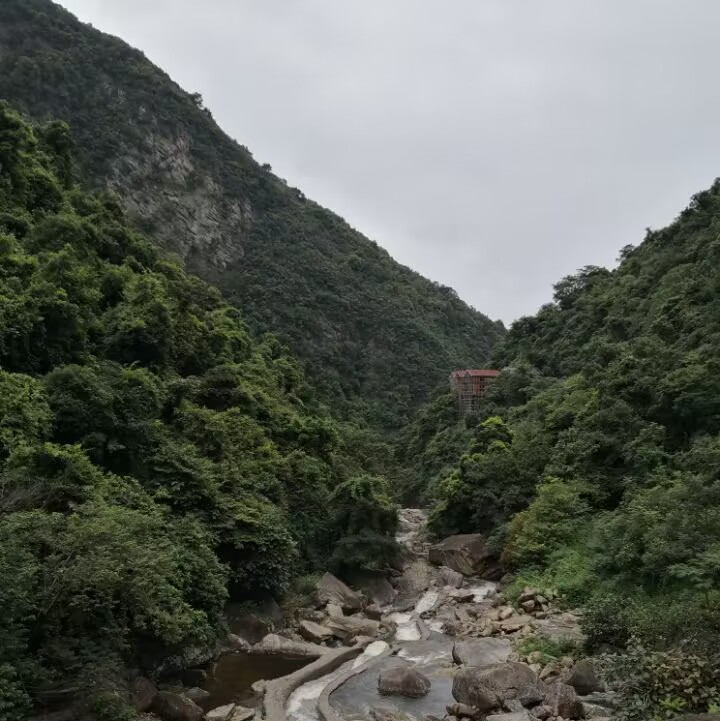
Damingshan (Wuming District) (Photo by Xiaohua Dai);

**Figure 2c.**
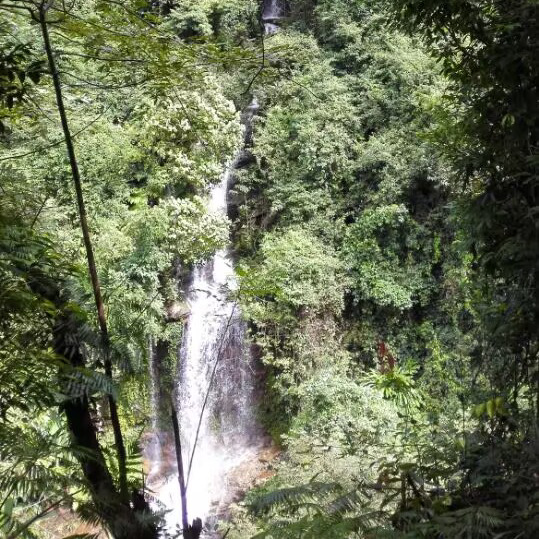
Damingshan (Mashan County) (Photo by Xiaohua Dai);

**Figure 2d.**
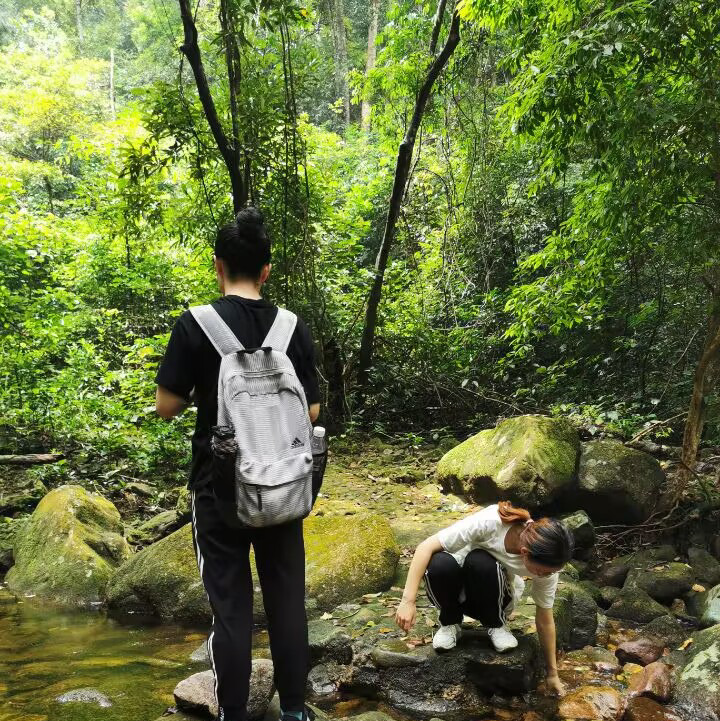
Shiwandashan (Photo by Xiaohua Dai).

**Figure 3a.**
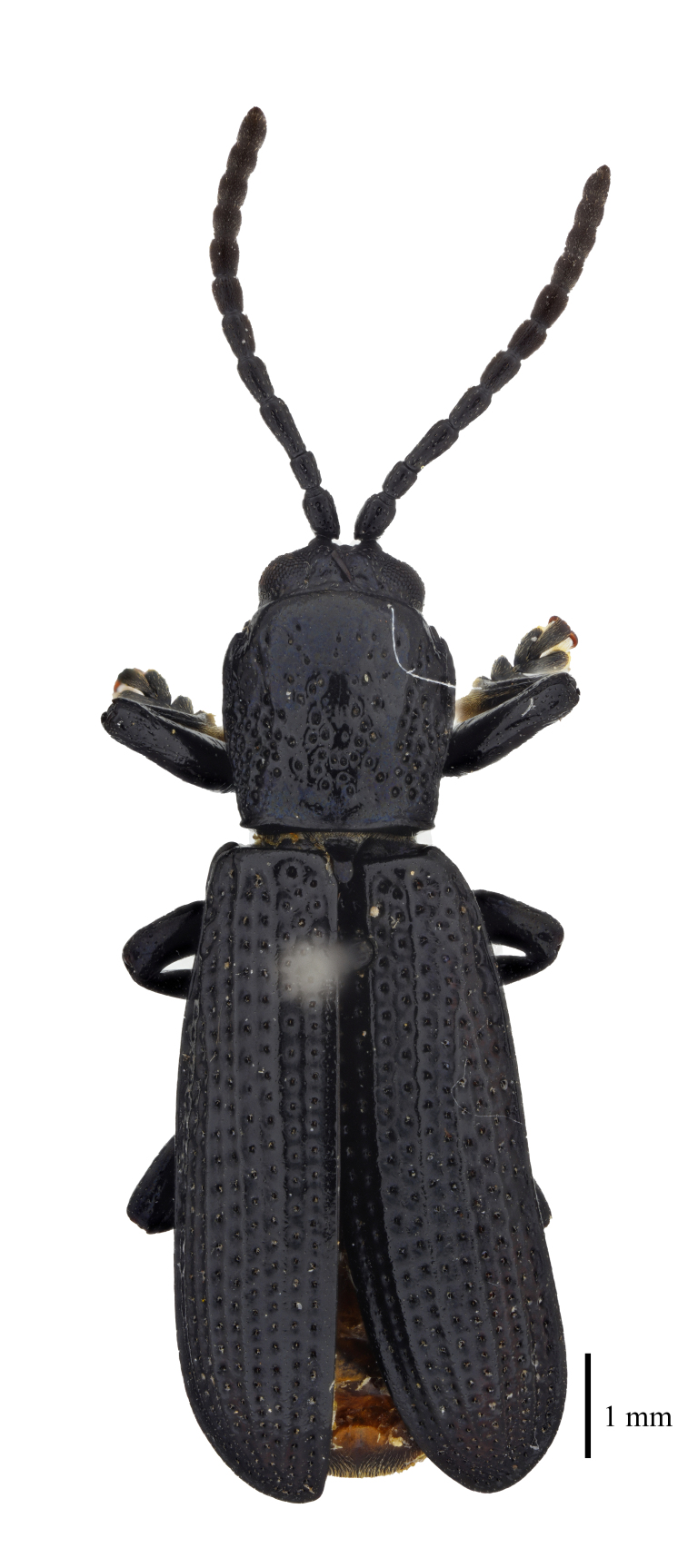
*Lasiochilaanthracina* Yu, 1985: Anisoderini;

**Figure 3b.**
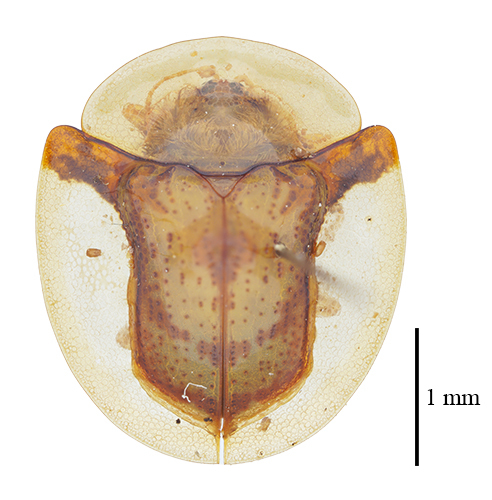
Aspidimorpha(s. str.)furcata (Thunberg, 1789): Aspidimorphini;

**Figure 3c.**
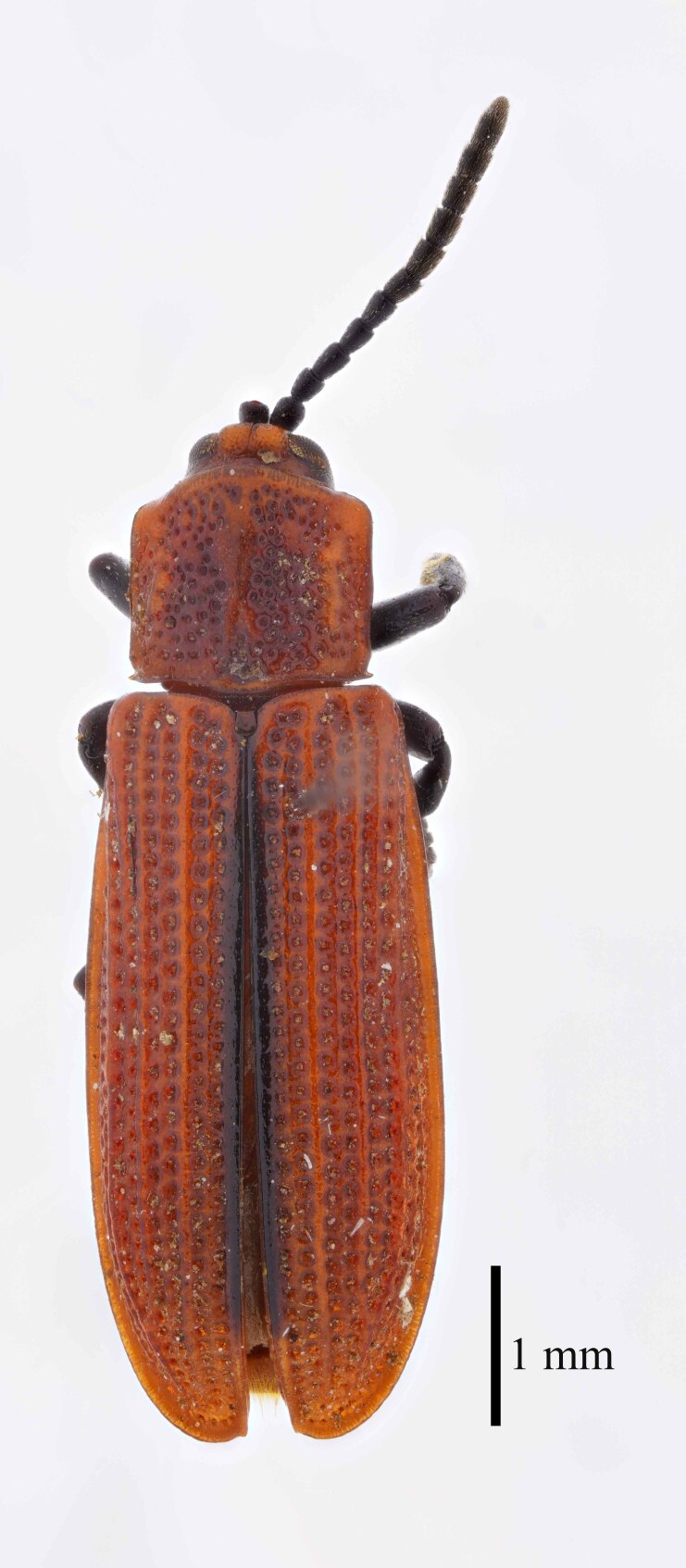
*Neodownesiarubra* Gressitt, 1953: Botryonopini;

**Figure 3d.**
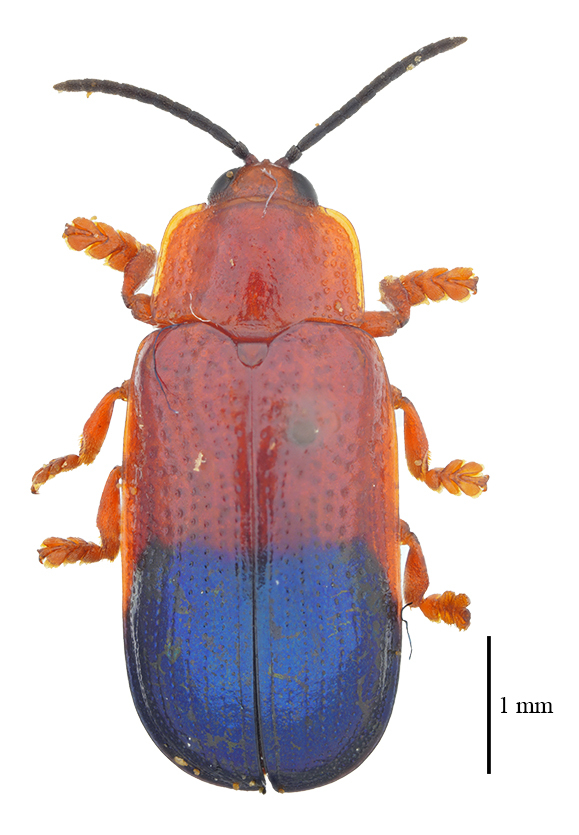
*Callispadimidiatipennis* Baly, 1858: Callispini;

**Figure 3e.**
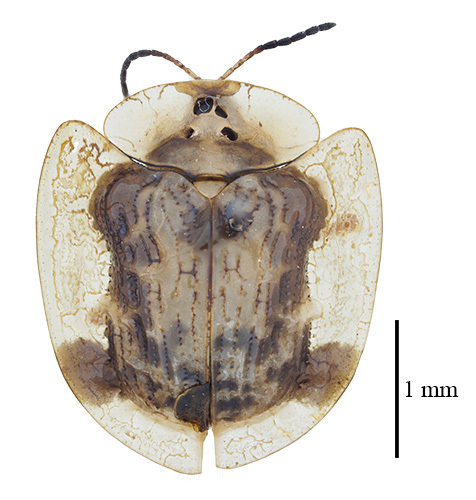
*Thlaspidabiramosa* (Boheman, 1855): Cassidini;

**Figure 3f.**
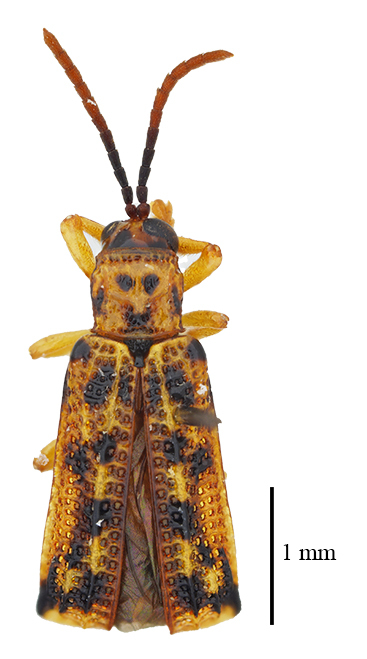
*Sinagoniafoveicollis* Chen et T'an, 1962: Gonophorini.

**Figure 4a.**
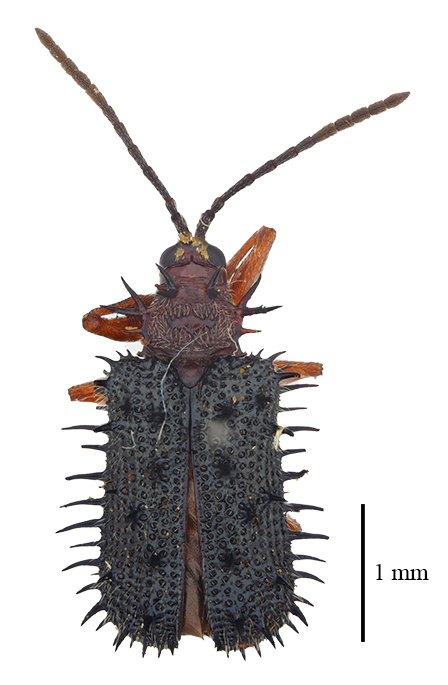
*Dactylispasetifera* (Chapuis, 1877): Hispini;

**Figure 4b.**
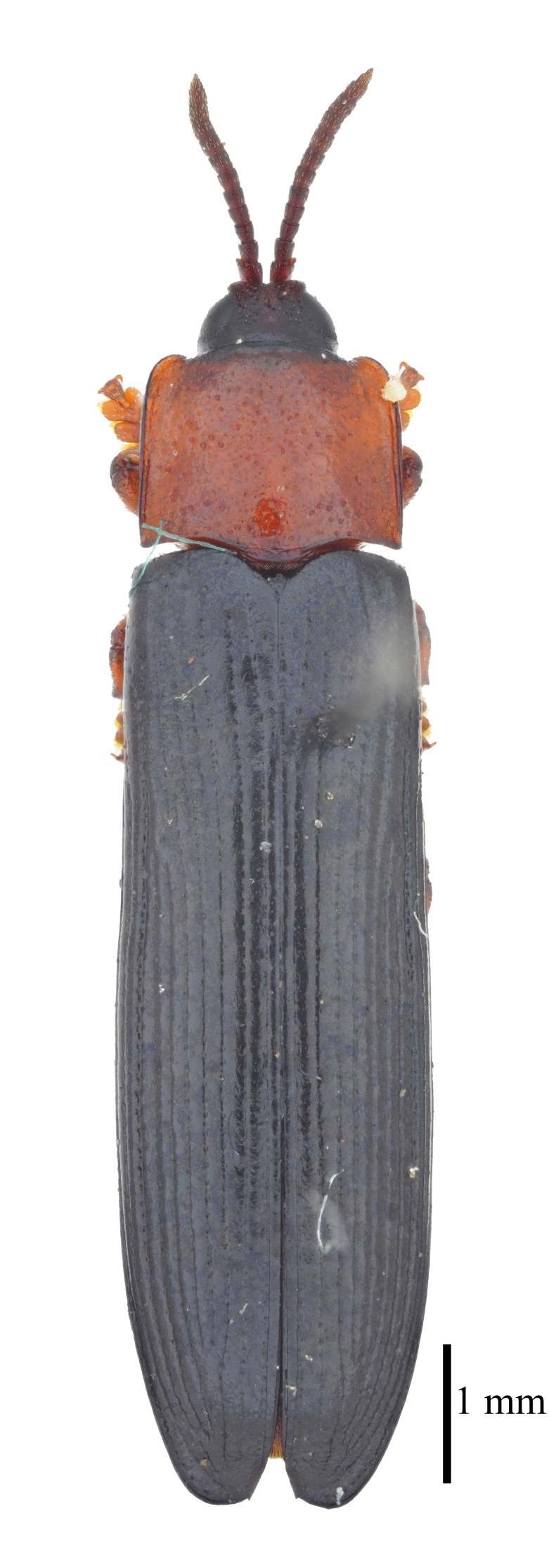
*Leptispalongipennis* Gestro, 1890: Leptispini;

**Figure 4c.**
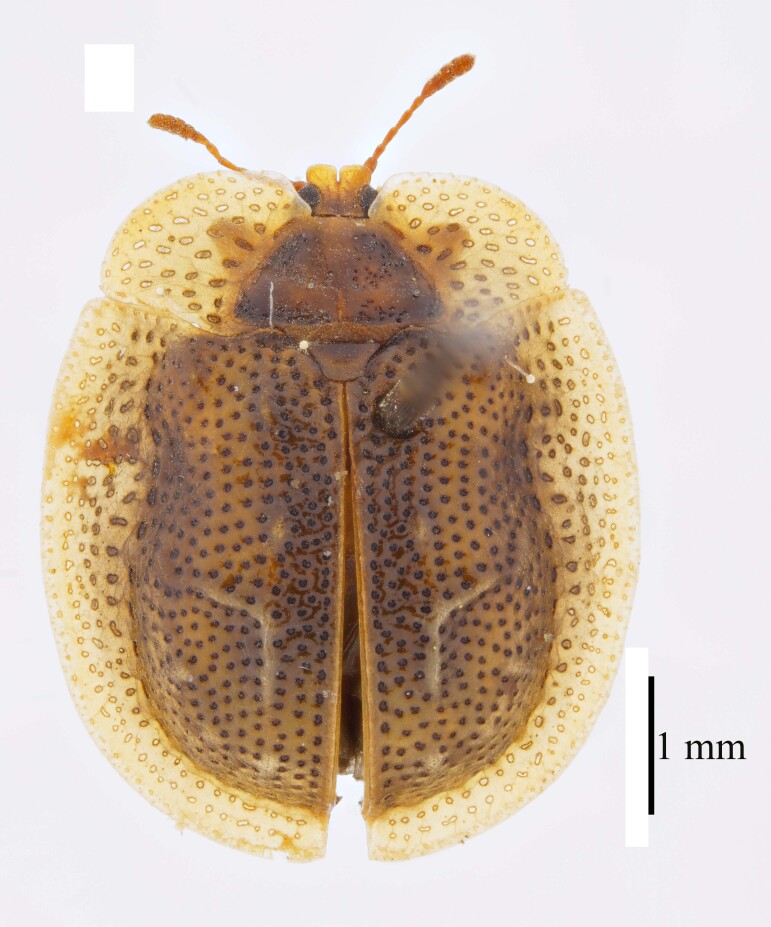
*Notosacanthasauteri* (Spaeth, 1914): Notosacanthini;

**Figure 4d.**
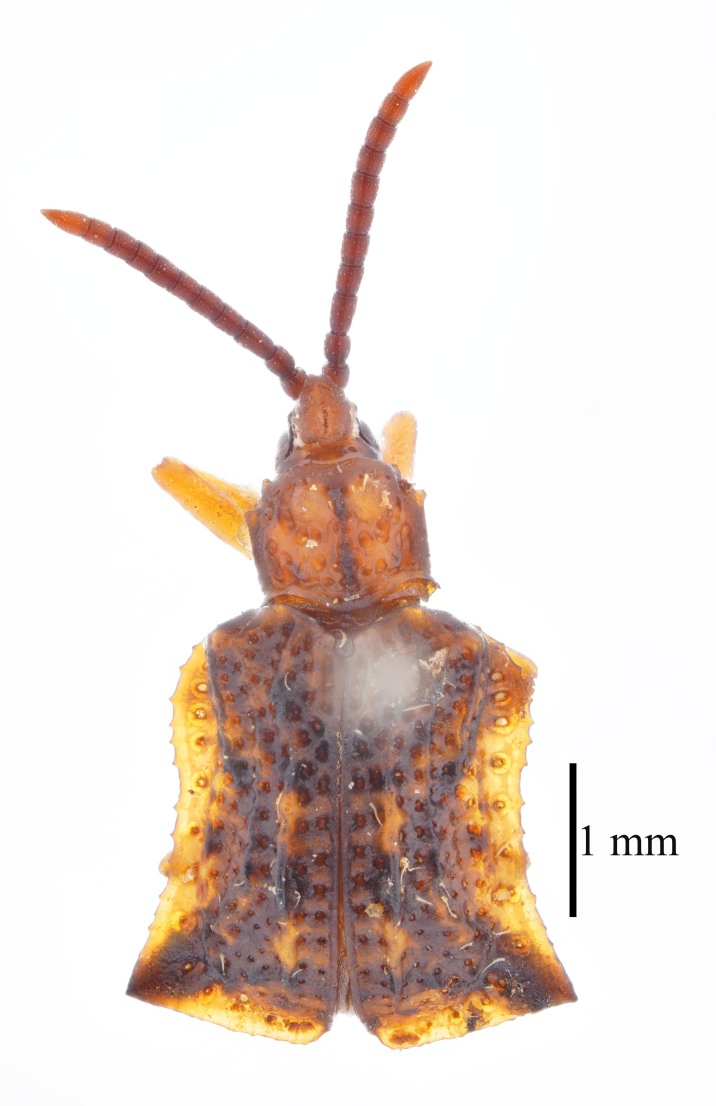
*Oncocephalahemicyclica* Chen et Yu, 1962: Oncocephalini.

**Table 1.** Quantitative food web metrics used in this study.

Bipartite network metrics	Description and meaning (Dormann et al. 2008, Dormann et al. 2009).
links per taxa	Average number of links per taxa.
connectance	Realised proportion of all possible links = standardised number of species combinations. Higher value indicates higher connectance.
generality	Mean effective number of host plant families per Cassidinae group.
vulnerability	Mean effective number of Cassidinae groups per plant family.
linkage density	Average of generality and vulnerability, indicating a weighted diversity of interactions per taxa.
specialisation degree *H*_2_’	*H*_2_’ indicates the degree of specialisation at network level, ranging between 0 (no specialisation) and 1 (complete specialisation): higher *H*_2_’ means higher specialisation.
weighted nestedness	A measure of network nestedness weighted by interaction frequencies, ranging between 0 (perfect chaos) and 1 (perfect nestedness). Higher nestedness means lower probability of extinction.
robustness	A measure of the network stability to taxa disappearance. Higher robustness indicates higher stability.

**Table 2.** Cassidinae beetles and their host plants in southern Guangxi. Note: "$" new record in China, "*" new record in Guangxi, "#" new record in southern Guangxi, "-" unknown or unidentified plant, "?" uncertain plant species, "&" new record of host plant species for the corresponding Cassidinae species.

Cassidinae beetles	Host plants
*Anisoderafraterna* Baly, 1888^#^	*Alpiniaoblongifolia Hayata^&^* *Zingibermioga* (Thunb.) Rosc.^&^
*Lasiochilaanthracina* Yu, 1985^#^	*Ampelocalamusactinotrichus* (Merr. et Chun) S. L. Chen et al.^&^ *Indocalamuslongiauritus* Handel-Mazzetti^&^ *Pseudosasaamabilis* (McClure) Keng f.^&^
*Lasiochilacylindrica* Hope, 1831	*Microstegiumfasciculatum* (Linnaeus) Henrard^&^ *Lophatherumgracile* Brongn.^&^ *Pseudosasaamabilis* (McClure) Keng f.^&^
*Aspidomorphacalligera* Boheman, 1854	Puerariamontanavar.lobata (Willdenow) Maesen & S. M. Almeida ex Sanjappa & Predeep^&^
*Aspidimorpha* (*s. str.*) *furcata* (Thunberg, 1789)	Merremiaumbellatasubsp.orientalis (H.Hallier) van Ooststroom
*Aspidimorpha* (*s. str.*) *miliaris* (Fabricius, 1775)	*Ipomoeacairica* (L.) Sweet
*Aspidimorpha* (*s. str.*) *sanctaecrucis* (Fabricius, 1792)	Merremiaumbellatasubsp.orientalis (H.Hallier) van Ooststroom^&^
Laccoptera (Laccopteroidea) nepalensis Boheman, 1855^#^	*Ipomoeabatatas* (L.) Lamarck Merremiaumbellatasubsp.orientalis (H.Hallier) van Ooststroom^&^
Laccoptera (Sindiola) vigintisexnotata Boheman, 1855^#^	-
*Neodownesiarubra* Gressitt, 1953^*^	*Carexcruciata* Wahlenb.^&^
*Callispa* sp. (near *C.apicalis* Pic, 1924)^*^	*Bambusaemeiensis* L. C. Chia & H. L. Fung^&^ *Pseudosasaamabilis* (McClure) Keng f.^&^
*Callispadimidiatipennis* Baly, 1858	*Indocalamuslongiauritus* Handel-Mazzetti^&^ *Indocalamustessellatus* (Munro) Keng f.^&^ *Miscanthussinensis* Anderss.^&^
*Callispaelliptica* Gressitt, 1939^*^	*Carexcruciata* Wahlenb.^&^
*Callispafrontalis* Medvedev, 1992^$^	*Indocalamuslongiauritus* Handel-Mazzetti^&^
*Callisparuficollis* Fairmaire, 1889^#^	*Fargesiaspathacea* Franch.^&^ *Indocalamuslongiauritus* Handel-Mazzetti^&^ *Pseudosasaamabilis* (McClure) Keng f.^&^
*Cassidacircumdata* Herbst, 1799	*Ipomoeabatatas* (L.) Lamarck *Ipomoeacairica* (L.) Sweet Merremiaumbellatasubsp.orientalis (H.Hallier) van Ooststroom^&^
*Cassidaconchyliata* (Spaeth, 1914)^*^	Merremiaumbellatasubsp.orientalis (H.Hallier) van Ooststroom^&^
*Cassidajapana* Baly, 1874^#^	*Ipomoeabatatas* (L.) Lamarck
*Cassidanucula* Spaeth, 1914^*^	*Alchorneatrewioides* (Benth.) Muell. Arg.^&^
*Cassidarati* Maulik, 1923^#^	*Brideliabalansae* Tutcher^&^
*Cassidaversicolor* (Boheman, 1855)^#^	-
*Chiridopsisbowringii* (Boheman, 1855)	Merremiaumbellatasubsp.orientalis (H.Hallier) van Ooststroom^&^
*Thlaspidabiramosa* (Boheman, 1855)^#^	*Callicarpabodinieri* Levl.^&^ *Callicarpakochiana* Makino *Callicarpamacrophylla* Vahl^&^
*Agonitachinensis* (Weise, 1922)	*Indocalamusbarbatus* McClure^&^ *Saccharumarundinaceum* Retz.^&^
*Agonitaimmaculata* (Gestro, 1888)^*^	*Indocalamusbarbatus* McClure^&^ *Indocalamustessellatus* (Munro) Keng f.^&^
*Agonitaindenticulata* (Pic, 1924)^*^	*Pseudosasaamabilis* (McClure) Keng f.^&^
*Agonitatricolor* Chûjô, 1933^*^	*Pleioblastusamarus* (Keng) Keng f.^&^
*Downesiaatrata* Baly, 1869^*^	*Carexcruciata* Wahlenb.^&^
*Downesiafulvipennis* Baly, 1888^*^	-
*Downesiaruficolor* Pic, 1924^*^	*Carexcruciata* Wahlenb.^&^ *Indocalamustessellatus* (Munro) Keng f.^&^
*Downesiatarsata* Baly, 1869^*^	*Carexcruciata* Wahlenb.^&^ *Digitariasanguinalis* (L.) Scop.^&^ *Indocalamusbarbatus* McClure^&^ *Microstegiumfasciculatum* (Linnaeus) Henrard^&^ *Miscanthusfloridulus* (Lab.) Warb. ex Schum et Laut. *Miscanthussinensis* Anderss.^&^
*Gonophorapulchella* Gestro, 1888^*^	*Alpiniakwangsiensis* T. L. Wu et Senjen^&?^
*Klitispamutilata* Chen et Sun, 1964^#^	*Carexcruciata* Wahlenb.^&^ *Digitariasanguinalis* (L.) Scop.^&^ *Miscanthussinensis* Anderss.^&^
*Klitisparugicollis* Gestro, 1890^*^	*Digitariasanguinalis* (L.) Scop.^&^
*Micrispadentatithorax* Pic, 1924^*^	*Alpiniaoblongifolia* Hayata^&^
*Sinagoniafoveicollis* Chen et T'an, 1962	*Cyperuscompressus* L.^&^ *Hypolytrumnemorum* (Vahl) Sprengel^&^ *Paspalumlongifolium* Roxb.^&^
*Asamangulialongispina* Gressitt, 1950^*^	*Miscanthussinensis* Anderss.^&^
*Dactylispaapproximata* Gressitt, 1939	*Setariapalmifolia* (koen.) Stapf^&^
*Dactylispabalyi* Gestro, 1890	*Digitariaciliaris* (Retz.) Koel.^&^ Ischaemumaristatumvar.glaucum (Honda) T.Koyama^&^ *Microstegiumvimineum* (Trin.) A. Camus^&^
*Dactylispachaturanga* Maulik, 1919^*^	*Sterculialanceolata* Cav.^&^
*Dactylispachinensis* Weise, 1905^#^	*Callicarpakochiana* Makino^&^ *Rubusalceifolius* Poiret *Rubuscaudifolius* Wuzhi^&^ *Rubuscochinchinensis* Tratt.^&^
*Dactylispacorpulenta* Weise, 1897^*^	*Byttneriagrandifolia* Candolle^&^ Antidesmamontanumvar.microphyllum (Hemsley) Petra Hoffmann^&^
*Dactylispafeae* Gestro, 1888	*Commelinapaludosa* Bl.^&^ *Arthraxonhispidus* (Trin.) Makino^&^ *Digitariasanguinalis* (L.) Scop.^&^ *Digitariaviolascens* Link^&^ *Indocalamusbarbatus* McClure^&^ *Isachneglobose* (Thunb.) Kuntze^&^ *Isachnetruncata* A. Camus^&^ Ischaemumaristatumvar.glaucum (Honda) T.Koyama^&^ *Lophatherumgracile* Brongn.^&^ *Microstegiumfasciculatum* (Linnaeus) Henrard^&^ *Miscanthussinensis* Anderss.^&^ *Miscanthusfloridulus* (Lab.) Warb. ex Schum et Laut.^&^ *Oplismenusundulatifolius* (Arduino) Beauv.^&^ *Ottochloanodosa* (Kunth) Dandy^&^ *Paspalumlongifolium* Roxb.^&^ *Setariageniculata* (Lam.) Beauv.^&^
*Dactylispaflavomaculata* Uhmann, 1930^*^	*Lophatherumgracile* Brongn.^&^
*Dactylispafukienica* Chen et T'an, 1964^*^	*Lophatherumgracile* Brongn.^&^
*Dactylispahigoniae* Lewis, 1896^#^	*Callicarpakochiana* Makino *Callicarpamacrophylla* Vahl^&^
*Dactylispaintermedia* Chen et T'an, 1961^*^	*Rubuscochinchinensis* Tratt.^&^
*Dactylispaklapperichi* Uhmann, 1954^*^	*Rubuscochinchinensis* Tratt.^&^
*Dactylispalameyi* Uhmann, 1930	-
*Dactylispalongispina* Gressitt, 1938	*Hypolytrumnemorum* (Vahl) Sprengel^&^ *Indocalamusbarbatus* McClure^&^ *Microstegiumfasciculatum* (Linnaeus) Henrard^&^ *Miscanthussinensis* Anderss.^&^ *Setariapalmifolia* (koen.) Stapf
*Dactylispamaculithorax* Gestro, 1906^*^	*Photiniabodinieri* Lévl.^&^
*Dactylispanigrodiscalis* Gressitt, 1938^#^	*Metadinatrichotoma* (Zoll. et Mor.) Bakh. F.^&^ *Mussaendapubescens* W. T. Aiton^&^ *Uncariarhynchophylla* (Miq.) Miq. ex Havil.^&^
*Dactylispapici* Uhmann, 1934^*^	*Elaeocarpusduclouxii* Gagnep.^&^
*Dactylispapilosa* T'an et Kung, 1961	Ischaemumaristatumvar.glaucum (Honda) T.Koyama^&^ *Microstegiumfasciculatum* (Linnaeus) Henrard^&^ *Microstegiumvimineum* (Trin.) A. Camus^&^
*Dactylispasauteri* Uhmann, 1927^#^	*Arthraxonprionodes* (Steudel) Dandy^&^ *Capillipediumassimile* (Steud.) A. Camus^&^ *Isachnetruncate* A. Camus^&^ *Lophatherumgracile* Brongn.^&^ *Miscanthussinensis* Anderss.^&^ *Pseudosasaamabilis* (McClure) Keng f.^&^ *Saccharumarundinaceum* Retz.^&^
*Dactylispasetifera* Chapuis, 1877	*Microstegiumfasciculatum* (Linnaeus) Henrard^&^ *Miscanthussinensis* Anderss.^&^ *Miscanthusfloridulus* (Lab.) Warb. ex Schum et Laut.^&^ *Thysanolaenalatifolia* (Roxburgh ex Hornemann) Honda^&^
*Dactylispauhmanni* Gressitt, 1950^*^	*Rubuscochinchinensis* Tratt.^&^
*Hispaandrewesi* Weise, 1897	*Microstegiumfasciculatum* (Linnaeus) Henrard^&^ *Miscanthussinensis* Anderss.^&^ *Miscanthusfloridulus* (Lab.) Warb. ex Schum et Laut.^&^ *Saccharumarundinaceum* Retz.^&^ *Isachneglobose* (Thunb.) Kuntze^&^
*Rhadinosafleutiauxi* Baly, 1889	*Digitariaviolascens* Link^&^ *Microstegiumfasciculatum* (Linnaeus) Henrard^&^
*Leptispacollaris* Chen et Yu, 1961^*^	*Miscanthussinensis* Anderss.^&^
*Leptispalongipennis* Gestro, 1890^#^	*Indocalamusbarbatus* McClure^&^
*Notosacanthasauteri* (Spaeth, 1914)^*^	*Rhododendroncavaleriei* Levl.^&^
*Notosacantha* sp. (near *N.trituberculata* Gressitt, 1952)^*^	*Ilexediticostata* Hu et Tang^&^ *Elaeocarpusglabripetalus* Merr.^&^ *Rhododendroncavaleriei* Levl.^&^ *Iteachinensis* Hook. et Arn.^&^ *Brideliabalansae* Tutcher^&^ *Aporosadioica* (Roxburgh) Muller Argoviensis^&^ *Lasianthuscurtisii* King et Gamble^&^ *Ophiorrhizakwangsiensis* Merr. ex Li^&^ *Pavettahongkongensis* Bremek.^&^ *Schizomussaendahenryi* (Hutch.) X. F. Deng et D. X. Zhang^&^ Schisandrapropinquasubsp.sinensis (Oliver) R. M. K. Saunders^&^ *Turpiniaarguta* (Lindl.) Seem.^&^
*Oncocephalahemicyclica* Chen et Yu, 1962^*^	Merremiaumbellatasubsp.orientalis (H.Hallier) van Ooststroom^&^
*Prionispaclavata* (Yu, 1992)^*^	-
*Prionispa* sp. (near *P.sinica* Gressitt, 1950)^*^	*Commelinapaludosa* Bl.^&^ *Pollia japónica* Thunb.^&^ *Helleniaspeciosa* (J.Koenig) S.R.Dutta^&^

**Table 3.** Characteristics of plant family-Cassidinae tribe food web and plant family-Cassidinae genus food web in southern Guangxi.

Bipartite network metrics	Host family-Cassidinae tribe	Host family-Cassidinae genus
number of plant taxa	19	19
number of Cassidinae taxa	10	23
links per taxa	1.207	1.095
connectance	0.184	0.105
generality	4.009	3.636
vulnerability	2.721	4.839
linkage density	3.365	4.238
specialisation degree *H*_2_’	0.443	0.329
weighted nestedness	0.490	0.638
robustness	0.518	0.585
